# Hemolysis in patients with Extracorporeal Membrane Oxygenation therapy for severe Acute Respiratory Distress Syndrome - a systematic review of the literature

**DOI:** 10.7150/ijms.50217

**Published:** 2021-02-18

**Authors:** Lorenz A. Materne, Oliver Hunsicker, Mario Menk, Jan A. Graw

**Affiliations:** 1 Department of Anesthesiology and Operative Intensive Care Medicine CCM/CVK, Charité - Universitätsmedizin Berlin, corporate member of Freie Universität Berlin, Humboldt-Universität zu Berlin, and Berlin Institute of Health.; 2 ARDS/ECMO Centrum Charité, Charité - Universitätsmedizin Berlin, Berlin, Germany.; 3 Berlin Institute of Health (BIH), Berlin, Germany.

**Keywords:** veno-venous ECMO, ARDS, cell-free hemoglobin, hemolysis, ECMO-system, pump head thrombosis

## Abstract

The Acute Respiratory Distress Syndrome (ARDS) is common in patients on the Intensive Care Unit and associated with significant mortality rates. In situations of severe respiratory insufficiency and failure of all possible conservative therapeutic approaches, veno-venous extracorporeal membrane oxygenation (VV ECMO) is used as a final option for temporary replacement of pulmonary function. ARDS as well as sepsis and VV ECMO treatment are all associated with intravascular hemolysis. The extent and relevance of intravascular hemolysis in the context of ARDS therapy is unclear. This systematic review aims to summarize the current evidence on the incidence and associated complications of intravascular hemolysis in adult patients with ARDS and treatment with VV ECMO. The databases MEDLINE, EMBASE and Web of Science were systematically searched and 19 publications fulfilled inclusion criteria. The incidence of hemolysis in patients with ARDS and treatment with VV ECMO ranged from 0 to 41% with survivors showing lower incidences and less severe hemolysis. A pump head thrombosis and high blood flows (≥3 l/min) as well as use of dual-lumen cannulas but not different pump models were associated with increased hemolysis. In conclusion, intravascular hemolysis in patients with ARDS and treatment with VV ECMO is a common and relevant complication that appears associated with increased mortality. Apart from ECMO hardware-settings, no additional possible causes for increased red cell breakdown such as disease severity, duration of ECMO therapy, or number and quality of red blood cell transfusions were investigated. Further research is needed to determine the origin and relevance of intravascular hemolysis in patients with ARDS and treatment with VV ECMO.

## Introduction

The severe Acute Respiratory Distress Syndrome (ARDS), a disease of all age groups and associated with a high mortality, leads to death in 46% of cases with conventional therapy 1, 2. Although data are still poor, veno-venous extracorporeal membrane oxygenation (VV ECMO) has become a frequent rescue therapy in recent years 2-5. Therapy with VV ECMO secures oxygenation and decarboxylation and facilitates a lung-protective ventilation strategy to buy time for the recovery of the lungs or as a bridge to a lung transplant 6-8. During VV ECMO therapy venous blood is drained through a large bore cannula into an extracorporeal circuit. A pump drives the blood through a membrane oxygenator and finally oxygenated and decarboxylated blood is infused back into a large vein of the circulation 8.

Intravascular hemolysis is a known side effect of blood circulating through extracorporeal systems and membrane oxygenators 5. Current literature reports an incidence of approximately 18% in patients treated with ECMO. However, these data include veno-venous and veno-arterial ECMO-systems (VA ECMO) 5. There are only few studies reporting the incidence of hemolysis and potential hemolysis-associated side effects in patients treated exclusively with VV ECMO. In addition, accumulating evidence suggests a role of hemolysis in sepsis and ARDS where an increased plasma concentration of cell-free hemoglobin might be associated with an increased mortality and adverse clinical effects 9-11. Plasma hemoglobin scavenges endothelial-derived nitric oxide (NO) thereby inducing vasoconstriction and concomitant hypertension 12. Furthermore, cell-free hemoglobin releases the highly reactive heme molecule, which oxidizes proteins and lipids, induces oxidative stress, and triggers proinflammatory signaling pathways 13, 14.

In this systematic review the current evidence on the incidence of hemolysis in adult patients with ARDS and treatment with VV ECMO is investigated. Furthermore, the evidence on potential associations between intravascular hemolysis, morbidity and mortality, ECMO-settings, and ECMO-hardware is analyzed.

## Materials and Methods

### Search strategy

We included all studies with data on hemolysis in patients treated with VV ECMO.

The databases MEDLINE (Medical Literature Analysis and Retrieval System Online) and Web of Science were systematically searched using the reference management software EndNote X7 (Thomson Reuters) on September 30, 2019. In addition, the database EMBASE (Excerpta Medica dataBASE) was searched using Ovid (Ovid Technologies) on the same day. Publications were screened by the following search terms: [“ECMO” OR “extracorporeal support” OR “extracorporeal life support” OR “ECLS” OR “extracorporeal membrane oxygenation”] AND [“plasma haemoglobin” OR “cell-free haemoglobin” OR “free haemoglobin” OR “haptoglobin” OR “hemolysis”]. Duplicates were detected and deleted by EndNote and manually.

### Inclusion and exclusion criteria

**Figure 1:**
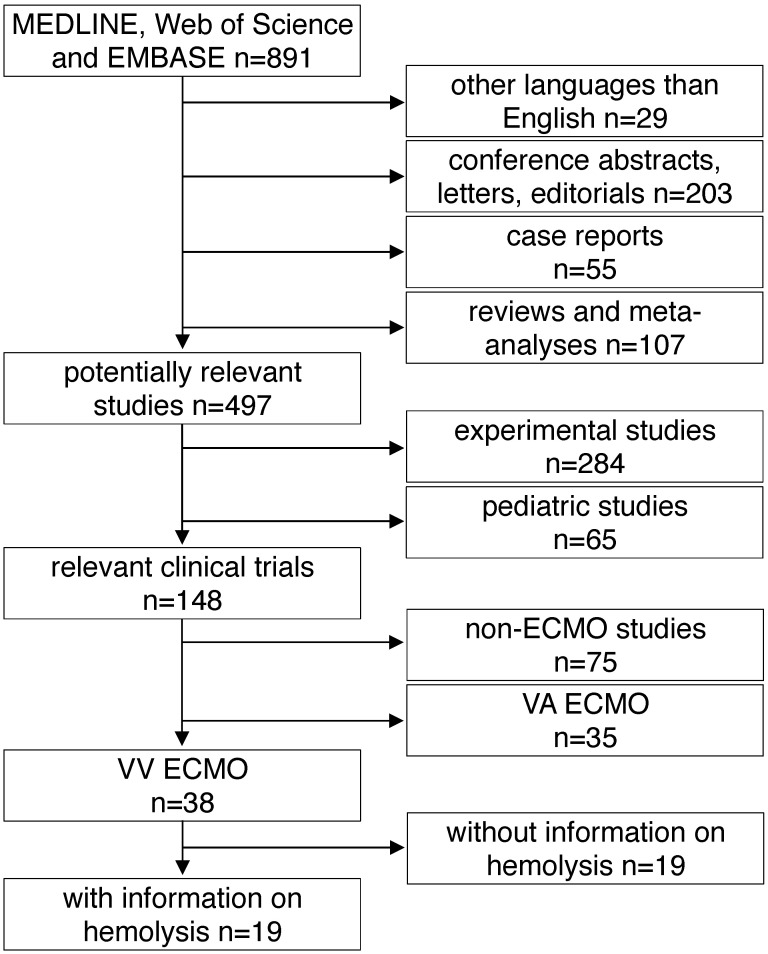
** Study flow diagram.** MEDLINE: Medical Literature Analysis and Retrieval System Online, EMBASE: Excerpta Medica dataBASE, VA ECMO: veno-arterial extracorporeal membrane oxygenation, VV ECMO: veno-venous extracorporeal membrane oxygenation.

All studies found by the above mentioned inclusion criteria were included into further analysis. The following exclusion criteria were applied in a top down hierarchic strategy (Fig. 1): 1) Case reports, comments, editorials, conference abstracts and reports, book chapters and letters to the editor were excluded from further analysis. Reviews, meta-analyses and all non-English papers were also excluded. 2) All non-human, experimental studies were excluded. 3) Studies in pediatric patients (patients aged <18 years) were excluded. 4) Studies on extracorporeal systems other than ECMO such as hemodialysis and cardiopulmonary bypass were excluded. 5) Studies including only treatment with VA ECMO were excluded. 6) Finally, all publications not providing information on hemolysis specifically in VV ECMO patients were excluded. Decisions were made based on the title and the abstract of each study meeting the search terms. For all studies where a clear decision was impossible after reading the abstract, the full text was analyzed. Furthermore, in unclear cases, decisions were made only after agreement and discussion between two independent reviewers.

### Data extraction

Full texts of all included studies were analyzed. For studies including patients with VV ECMO and VA ECMO therapy, characteristics of the whole study population treated with ECMO were discussed. If not provided in the papers, the following values were calculated from reported data: percentage of male patients, underlying disease and indication for ECMO therapy, proportion of patients with VV ECMO therapy, pump model, mode of cannulation, pump head thrombosis, and time on ECMO. If possible, incidence of hemolysis was reported or calculated. All data on fHb-measurements were converted to mg/dl. In addition, ECMO circuit characteristics such as pump flow, details of cannulas, pump model, and occurrence of pump head thrombosis were analyzed.

### Assessment of systematic reporting

The included studies were screened for information on systematic reporting strategies such as STROBE or CONSORT 15, 16.

## Results

A total of 891 articles met the search criteria and were screened in detail (Fig. 1). There were 497 original articles with 284 papers describing findings of non-clinical, experimental studies and 65 studies that were exclusively performed in pediatric patients. Thirty-eight of the remaining 148 clinical studies reported data on veno-venous extracorporeal organ support. Of these 38 studies, 19 studies reported data on hemolysis in patients treated with VV ECMO including data of 7,707 VV ECMO applications (Table 1).

### Basic study characteristics

Table 1 gives an overview of the 19 studies that were included in this systematic review. There were only two randomized, prospective but non-blinded trials including a total of 64 patients 17, 18. Data collection of all other studies occurred retrospectively. Chimot and colleagues used a survey to collect retrospective data and attained responses from 18 out of the 19 contacted institutes 19. Of the included studies only Fisser et al. stated that they made use of a systematic reporting strategy and provided a STROBE statement 20.

Seventeen of the 19 included studies represent a total of 7,350 patients ranging from 7 to 4,988 patients per study. The two other studies reported data on ECMO runs (n = 769) 19, 21. There were fife studies that included patient populations treated with both, va and VV ECMO 21-25. Fourteen studies analyzed patient cohorts that were exclusively treated with VV ECMO 17-20, 26-35.

### Incidence of hemolysis during therapy with VV ECMO

In fourteen of the included studies, hemolysis was quantified using plasma concentrations of cell-free hemoglobin (fHb) (Table 1) 17, 18, 20, 23-25, 27-33, 35. In addition, six of the studies reported plasma activity of lactate dehydrogenase (LDH) 17, 18, 23, 25, 30, 31. To quantify hemolysis, Bosma et al. defined a hemolysis-index (H-index) that significantly correlated with plasma levels of fHb 25. The H-index was automatically measured with every clinical chemistry blood analysis by absorbance measurements of the blood sample at different wavelengths by high throughput analyzers used in the authors' institution 25.

Six of the included studies did not report the incidence of hemolysis 17, 18, 20, 23, 29, 34. Moreover, it was not possible to calculate the incidence of hemolysis from the published data. In the remaining studies the reported or calculated hemolysis-incidence ranged from 0 to 41% (Table 2).

The two prospective, randomized studies were among the studies not reporting the incidence of hemolysis. However, Malfertheiner et al. noted a median fHb-plasma concentration of 54 mg/dl on the last day of ECMO therapy 17. On the day after ending ECMO therapy, plasma concentrations of fHb and LDH-activity decreased significantly 17. The second prospective, randomized study by Deatrick and colleagues reported normal fHb plasma concentrations but elevated LDH-activity without clear quantification 18. The retrospective study by Fisser et al. stated a mean concentration of fHb >50 mg/dl during ECMO treatment in both of the analyzed groups 20. Many of the included authors recognize measurements of fHb-concentration >50 mg/dl to define hemolysis 27, 28, 32, 35.

### Hemolysis and mortality

The studies of Kutlesa et al. and Omar et al. found a higher incidence of hemolysis in non-survivors (80-83%) compared to survivors (0-18%) (Table 2) 23, 28. From the data published by Chan et al. and Petersen et al. higher incidences of hemolysis could be calculated for non-survivors compared to survivors 22, 26. Similarly, in 2015 Lehle et al. reported that the average maximum plasma concentration of cell-free hemoglobin was significantly higher in non-survivors compared to survivors 31. The remaining studies did not report data on mortality in patients treated with VV ECMO with regard to increased plasma concentrations of cell-free hemoglobin (Table 2).

### Hemolysis and pump head thrombosis

Table 3 shows the studies that report pump head thromboses of the ECMO-system and their association with hemolysis. In 2014, Lehle and colleagues described a patient who had a fHb-plasma concentration greater than 100mg/dl caused by a pump head thrombosis 29. Furthermore, in 2015, the same group reported 8 pump head thromboses that were associated with hemolysis 31. Of note, after exchange of the pump head the increased plasma concentration of cell-free hemoglobin decreased within two days 31. Lubnow and colleagues reported 13 pump head thrombosis, all associated with an increase of hemolysis parameters cell-free hemoglobin and LDH-activity 30. In 6 of the patients the plasma concentration of cell-free hemoglobin was already elevated on the day before the pump head thrombosis occurred 30. Pan and colleagues reported five events of a pump head thrombosis which all was associated with hemolysis and occurred exclusively during therapy with VV ECMO 24. Bosma et al. reported a rise of the H-index when a pump head thrombosis occurred 25.

### Hemolysis and blood flow, cannulation and ECMO-system

Lehle and coworkers reported in 2014 that they could not find a causal link between an increase in blood flow and an increase in the plasma concentrations of cell-free hemoglobin 29. However, in 2015 the same working group reported a minimal but significant increase in fHb-plasma concentrations when blood flows ≥3 l/min were applied (Table 3) 31. Furthermore, fHb-plasma concentrations were greater in patients treated with dual-lumen NovaPort-24Ch-cannulas compared to larger dual-lumen cannula models (Table 3) 31. Mazzeffi et al. investigated patients treated with 27 and 31Ch dual-lumen cannulas showing no significant difference in fHb-plasma concentrations 35.

There was no association between increased hemolysis and the usage of a specific ECMO-system (Table 3). Of note, the diagonal pump Deltastream of Medos operating with a rotational speed twice as high compared to centrifugal pumps did not cause a relevant increase of fHb-plasma concentrations 17, 29, 31. All but one working group of those who reported data on the pump models that were used, utilized second-generation centrifugal pumps. Solely Guirand and colleagues applied roller pumps 27.

## Discussion

This systematic review includes 19 publications on adult ARDS patients treated with VV ECMO and associated intravascular hemolysis. The reported incidence of hemolysis varied greatly among the different studies. Hemolysis occurred more frequently in non-survivors compared to survivors of an ARDS and therapy with VV ECMO 22, 23, 26, 28, 31. All reported cases of a pump head thrombosis were accompanied by hemolysis 24, 25, 28-31, 34. The use of centrifugal- or diagonal-pump-systems had no, a high blood flow (≥3 l/min) had only a minimal effect on the occurrence of hemolysis 17, 29, 31. However, using a dual-lumen NovaPort-24Ch-cannula with high blood flow was associated with a significantly higher rate of hemolysis compared to the use of larger cannulas 31.

There were only two studies with a prospective study design 17, 18. Therefore, there is only a relatively low level of evidence describing an association between hemolysis and therapy with VV ECMO and it is unclear whether the ECMO is the reason for the damage of the red blood cells. In addition, the survey of Chimot and colleagues might suffer from a recall bias since data collection was done retrospectively 19.

The prospective study performed by Deatrick et al. only included ten patients. Because it was a pilot study, the authors did not perform a sample size calculation 18. Malfertheiner and colleagues expected an adequate statistical power with a sample size of 18 patients per group. Nevertheless, a statistical power calculation was not performed a priori. In addition, inclusion criteria required a minimum duration of five days of therapy with VV ECMO. However, at least one patient of one 18-patient cohort received VV ECMO only for four days 17.

Kurtlesa and colleagues published an exceptionally high hemolysis incidence of 41%, which might be influenced by the small number of included patients (n=17) 28. With a hemolysis incidence of 5% the recently publishes EOLIA-trial is within the common range 4. However, hemolysis was defined differently among the studies: A significant number of studies did not define a critical threshold for hemolysis or did not report any data on fHb-concentrations 19-22, 26, 29, 34. Whether the blood hemoglobin concentration or transfusion requirements are helpful parameters to estimate the degree of hemolysis remains questionable 19. In addition, transfusion of red blood cells itself can increase circulating levels of fHb especially when packed red blood cells are transfused that are near the end of the allowed storage duration 36, 37.

The search strategy of this systematic review considered all work published in MEDLINE, Web of Science and EMBASE. Because almost all included studies followed a retrospective study design, this review is limited to only report an association between hemolysis and therapy with VV ECMO but cannot claim a causal link between both entities. This systematic review is further limited by the heterogenic nature of the included studies. There was a wide variety of inclusion and exclusion criteria, sample sizes, time on and indication for ECMO, definition and quantification of hemolysis and measured endpoints. In most studies, male sex dominated the groups of included patients. Lubnow et al. included only patients that received a complete change of the ECMO-circuit during therapy with VV ECMO 30. Both studies of Lehle and colleagues excluded patients with hemolysis before ECMO therapy. However, the threshold for plasma concentrations of cell-free hemoglobin before therapy with VV ECMO was different in both studies 29, 31. Furthermore, patients with significant hemolysis during ECMO therapy were excluded when a cause for increased red blood cell break down such as a pump head thrombosis was considered evident 29, 31. For this systematic review the effects and strategies of anticoagulation during VV ECMO therapy that might have an impact on development of a pump head thrombosis were not analyzed. Additionally, it is known that hemolysis can be caused by high negative pressure within the drainage cannula 38. The negative pressure is linked to the small cannula size, high blood flow and patient specific factors. However, these connections were not investigated in this review. The study by Guirand et al. allowed inclusion of patients from the age of 16 27. Furthermore, from the data reported in the study by Hoshino et al. it is unclear whether truly only adults were included 34. Moreover, this review is limited by including different papers that accessed the same databases. Therefore, several papers may have reported data on the same set of patients 17, 20, 21, 29-32, 35. A meta-analysis of the studies with data from patients treated with VV ECMO and with a unique definition and severity grading of hemolysis might be necessary to obtain reliable information on the incidence of hemolysis during therapy with VV ECMO and whether hemolysis during therapy with VV ECMO might affect mortality in patients with severe ARDS.

Taken together, this systematic review indicated that hemolysis might be a frequent complication associated with VV ECMO therapy in severe ARDS 19, 24-26, 28, 30, 33. Nevertheless, especially the studies providing data on large VV ECMO populations reported incidences of hemolysis of less than 10% 21, 32, 35. In addition, there is emerging evidence that hemolysis might be associated with increased mortality during therapy with VV ECMO 22, 23, 26, 28, 31. It seems that in today's common VV ECMO-systems the pump model, cannulas and blood flow do not have a significant impact on the development of a hemolysis 17, 29, 31. On the other hand, various systemic diseases like ARDS or sepsis are associated with a certain degree of hemolysis 9, 10, 39. Whether hemolysis associated with VV ECMO therapy increases the side effects of fHb on systemic inflammation and end organ damage or whether therapy of the underlying disease using VV ECMO helps to reduce the total amount of circulating intravascular cell-free hemoglobin is unclear.

A prospective study that includes measurements for fHb-levels before the start of VV ECMO therapy, the quality and number of transfused red blood cell concentrates, and additional hemolysis markers such as haptoglobin or lactate dehydrogenase activity would be warranted. Such a study would clarify if VV ECMO-induced hemolysis clearly exceeds the levels of fHb that are released in patients with ARDS but not treated with ECMO. In addition, a prospective trial could also address whether hemolysis is just a marker of disease severity and mortality in patients with ARDS and VV ECMO therapy or whether hemolysis and the toxic effects of fHb might be relevant cofactors for disease progression. For the latter, treatment options could be developed that include scavenging and reduction of increased plasma concentrations of cell-free hemoglobin.

## Figures and Tables

**Table 1:**
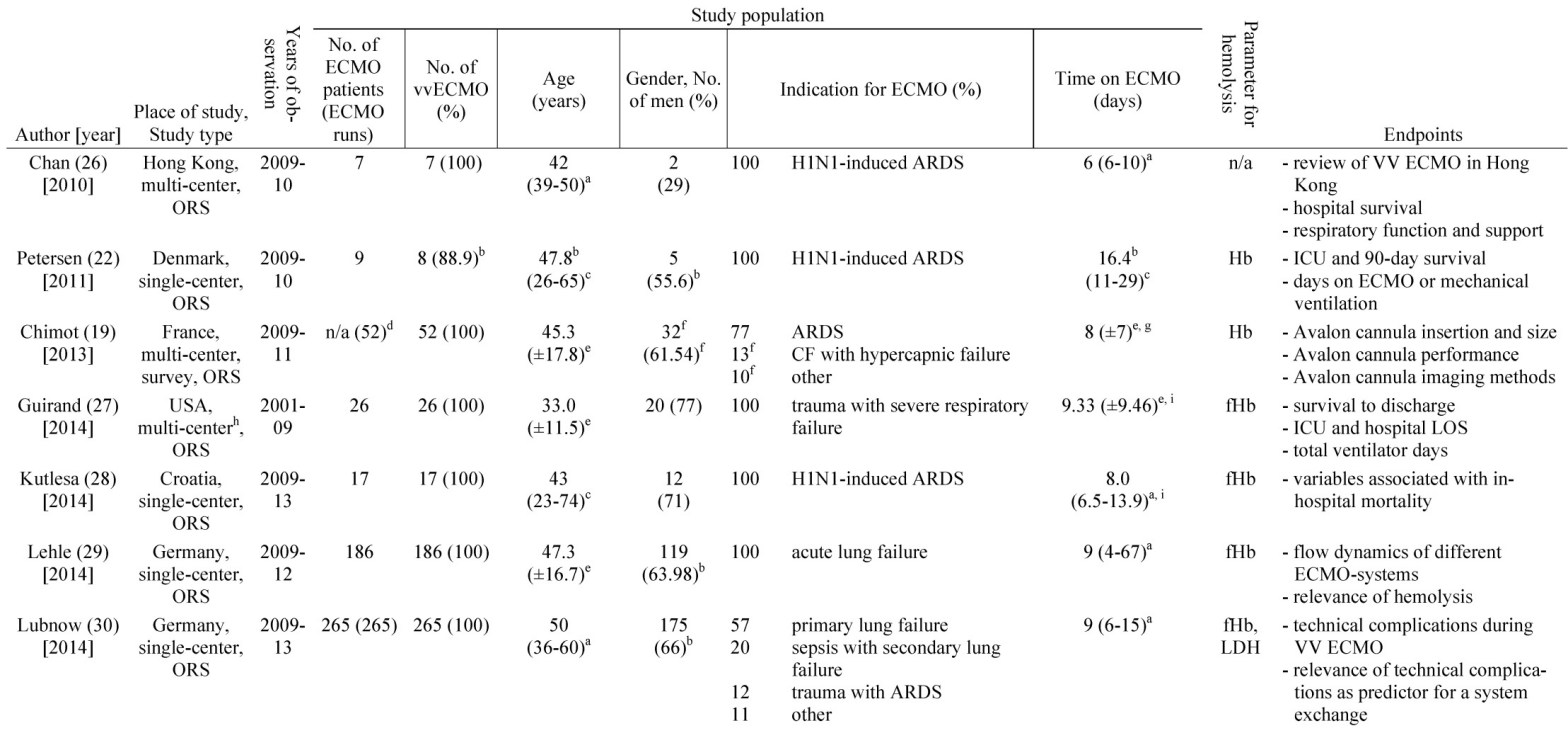

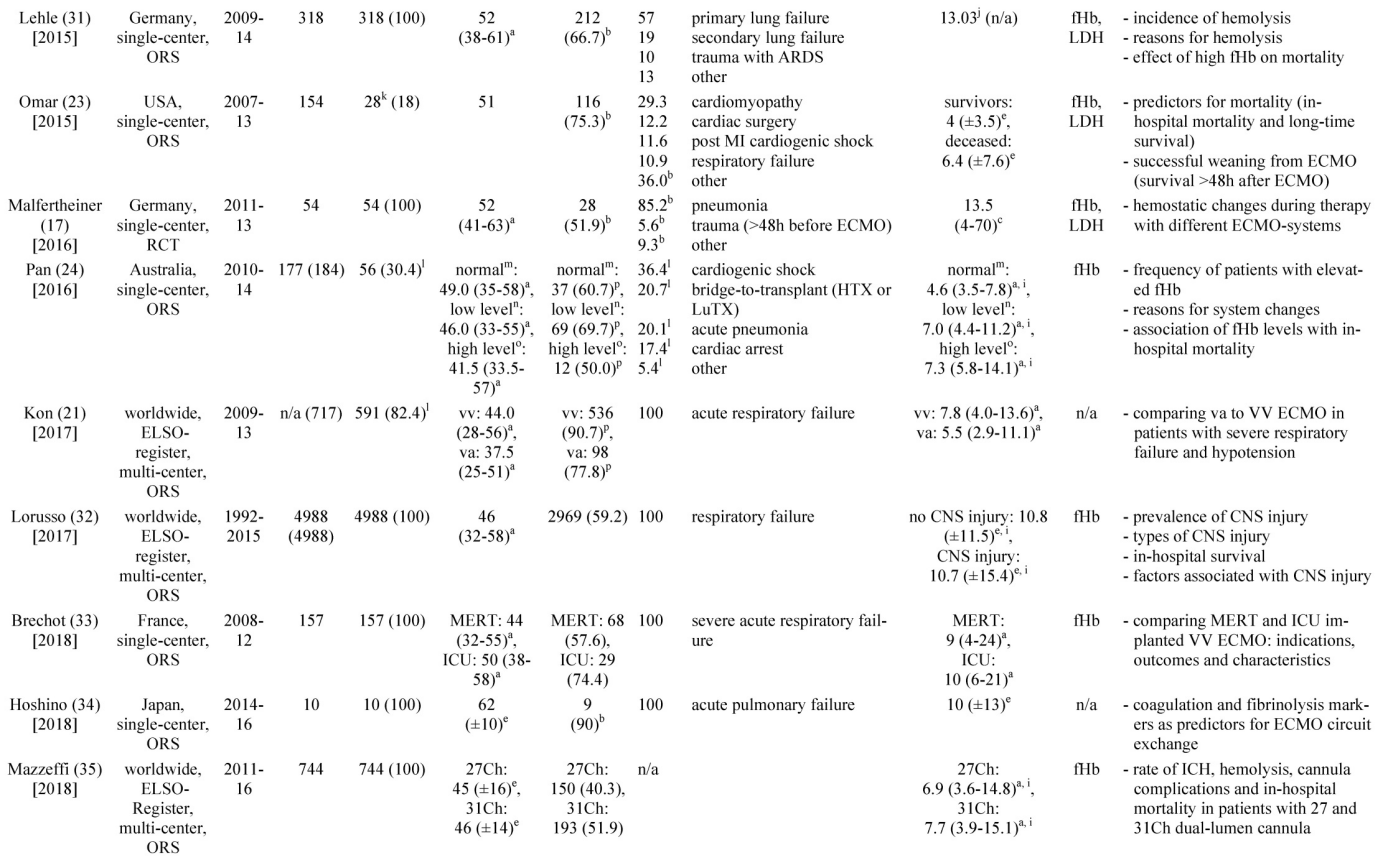

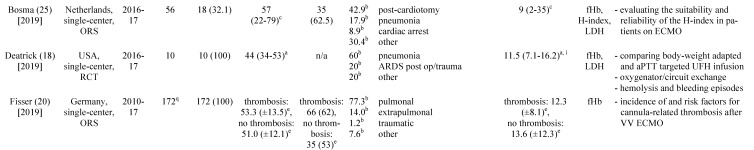
Characteristics of the included studies

**a**: interquartile range, **b**: mean or percentage calculated for the given number of ECMO patients, **c**: range, **d**: 52 cannula uses were reported, **e**: standard deviation, **f**: percentage calculated for 52 cannula uses, **g**: time on cannula, **h**: all ECMO Patients were treated in one hospital, **i**: days were calculated from hours, **j**: mean calculated from the given data for 318 patients, **k**: number calculated from the given percentage of vvECMO patients, **l**: percentage calculated for the given number of ECMO runs, **m**: fHb-plasma level <10mg/dl (n=61), **n**: fHb-plasma level 10-50mg/dl (n=99), **o**: fHb-plasma level >50mg/dl (n=24), **p**: percentage calculated for the number of ECMO runs in this subgroup, **q**: number of patients screened for venous thrombosis **ECMO**: extracorporeal membrane oxygenation, **VV ECMO**: veno-venous ECMO, **ORS**: observational retrospective study, **H1N1**: influenza virus subtype, **ARDS**: acute respiratory distress syndrome, **n/a**: not applicable, **Hb**: hemoglobin, **ICU**: intensive care unit, **CF**: cystic fibrosis, **Avalon cannula**: dual-lumen cannula in vvECMO, **fHb**: plasma free hemoglobin, **LOS**: length of stay, **LDH**: lactate dehydrogenase, **MI**: myocardial infarction, **RCT**: randomized controlled trial, **HTX**: heart transplant, **LuTX**: lung transplant, **ELSO**: Extracorporeal Life Support Organization, **va**: veno-arterial, **CNS**: central nervous system, **MERT**: mobile ECMO retrieval team, **Ch**: Charrière, **ICH**: intracranial hemorrhage, **H-index**: hemolysis index, **aPTT**: activated Partial Thrombo-plastin Time, **UFH**: unfractionated heparin

**Table 2:**
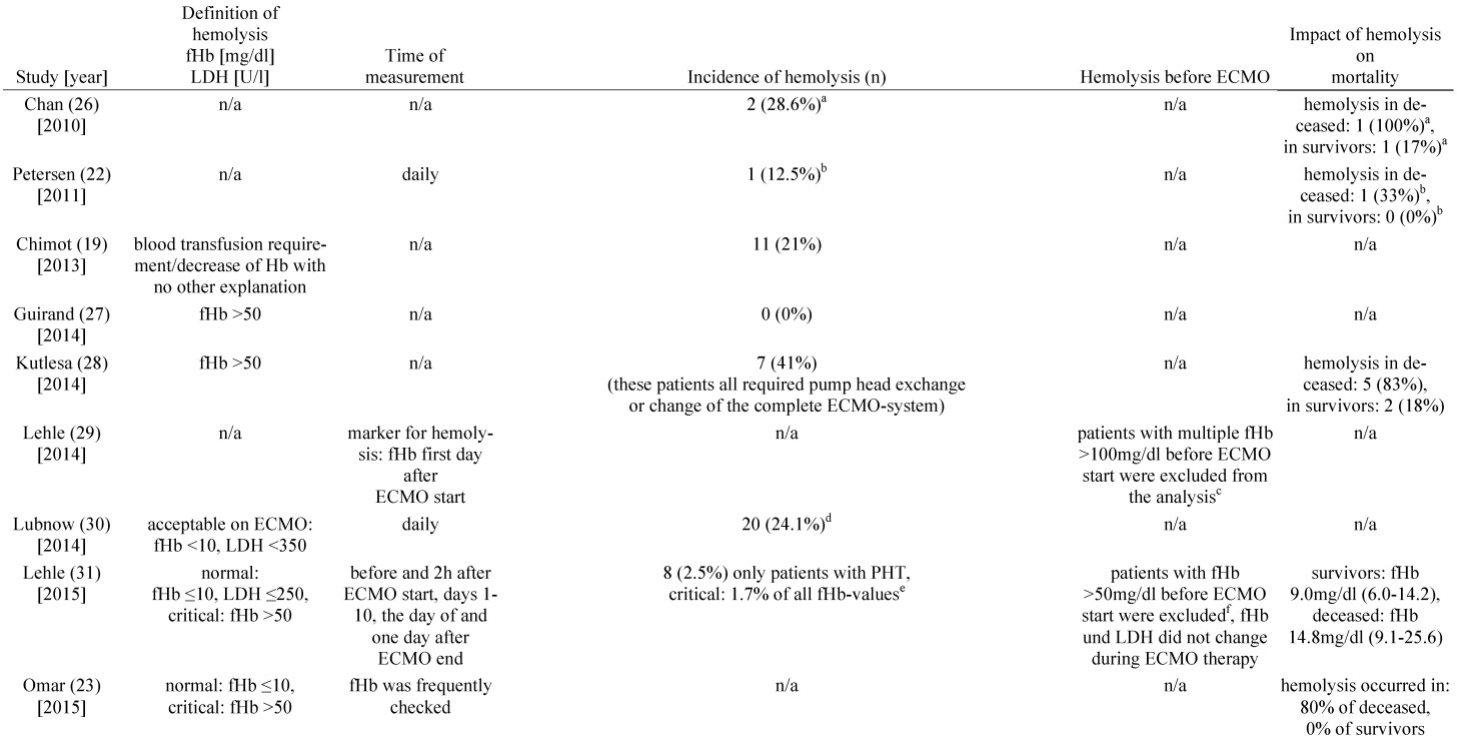

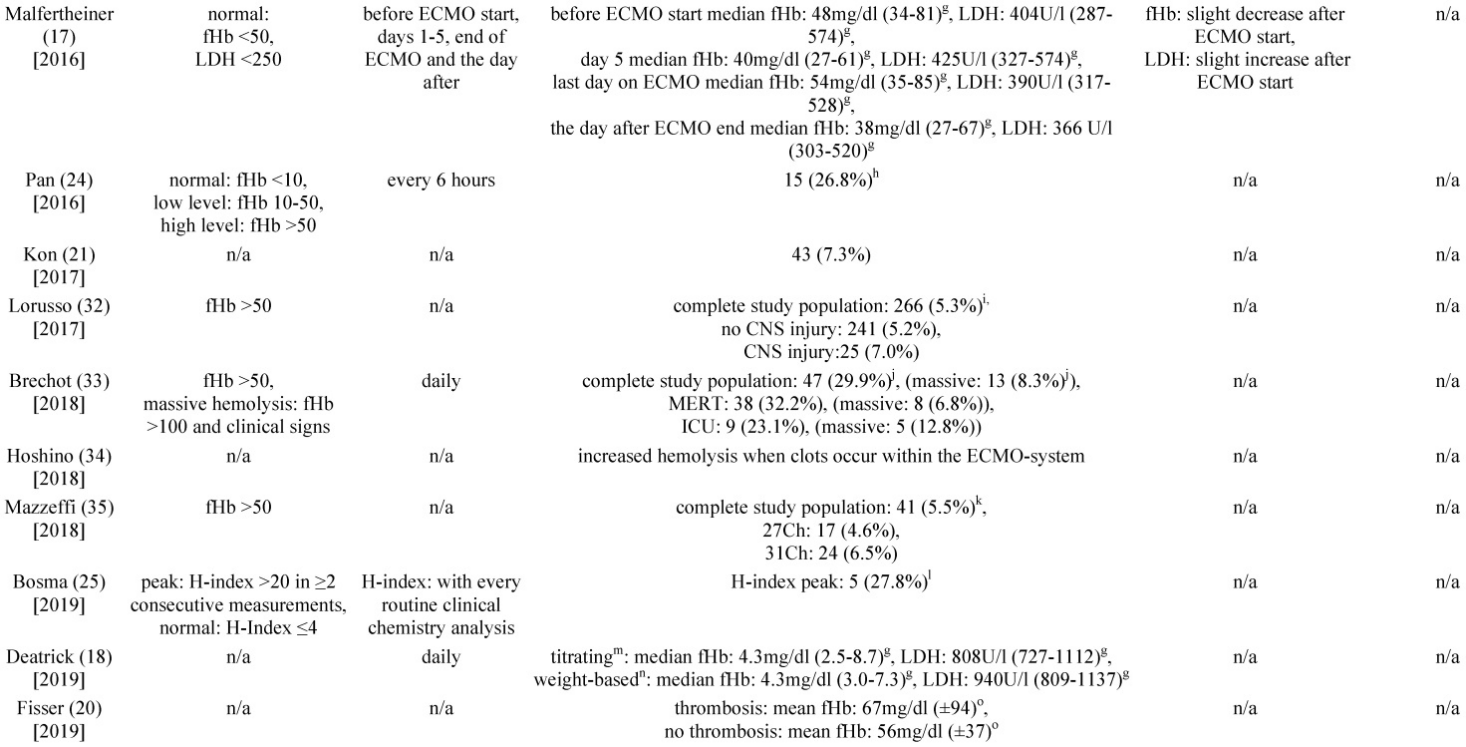
Incidence of hemolysis in VV ECMO

a: incidence calculated with the given figures for 7 patients, b: incidence calculated with the given figures for 8 patients, c: cause of elevated fHb: sepsis (n=24), massive transfusion (n=10), surgery on ECMO (n=5), PHT (n=1), clot within cannula (n=3), d: 20 cases of a rise in fHb were reported: PHT (n=13), clot in the membrane oxygenator (n=7), incidence calculated for 83 patients with system exchange, e: 4142 measurements were performed in total, f: sepsis (n=4), trauma (n=4), cardiac surgery (n=3), g: interquartile range, h: incidence calculated for 56 vvECMO runs, i: inci-dence calculated with the given figures for 4988 patients, j: incidence calculated with the given figures for 157 patients, k: incidence calculated with the given figures for 744 patients, l: incidence calculated for 18 vvECMO Patients, m: UFH targeting aPTT 45-55s, n: UFH body-weight adapted (10units/kg/h), o: standard deviation. **Abbreviations:** VV ECMO: veno-venous extracorporeal membrane oxygenation, fHb: plasma free hemoglobin, LDH: lactate dehydrogenase, n/a: not applicable, Hb: hemoglobin, PHT: pump head thrombosis, CNS: central nervous system, MERT: mobile ECMO retrieval team, ICU: intensive care unit, Ch: Charrière, H-index: hemolysis index, UFH: unfractionated heparin, aPTT: activated Partial Thromboplastin Time.

**Table 3:**
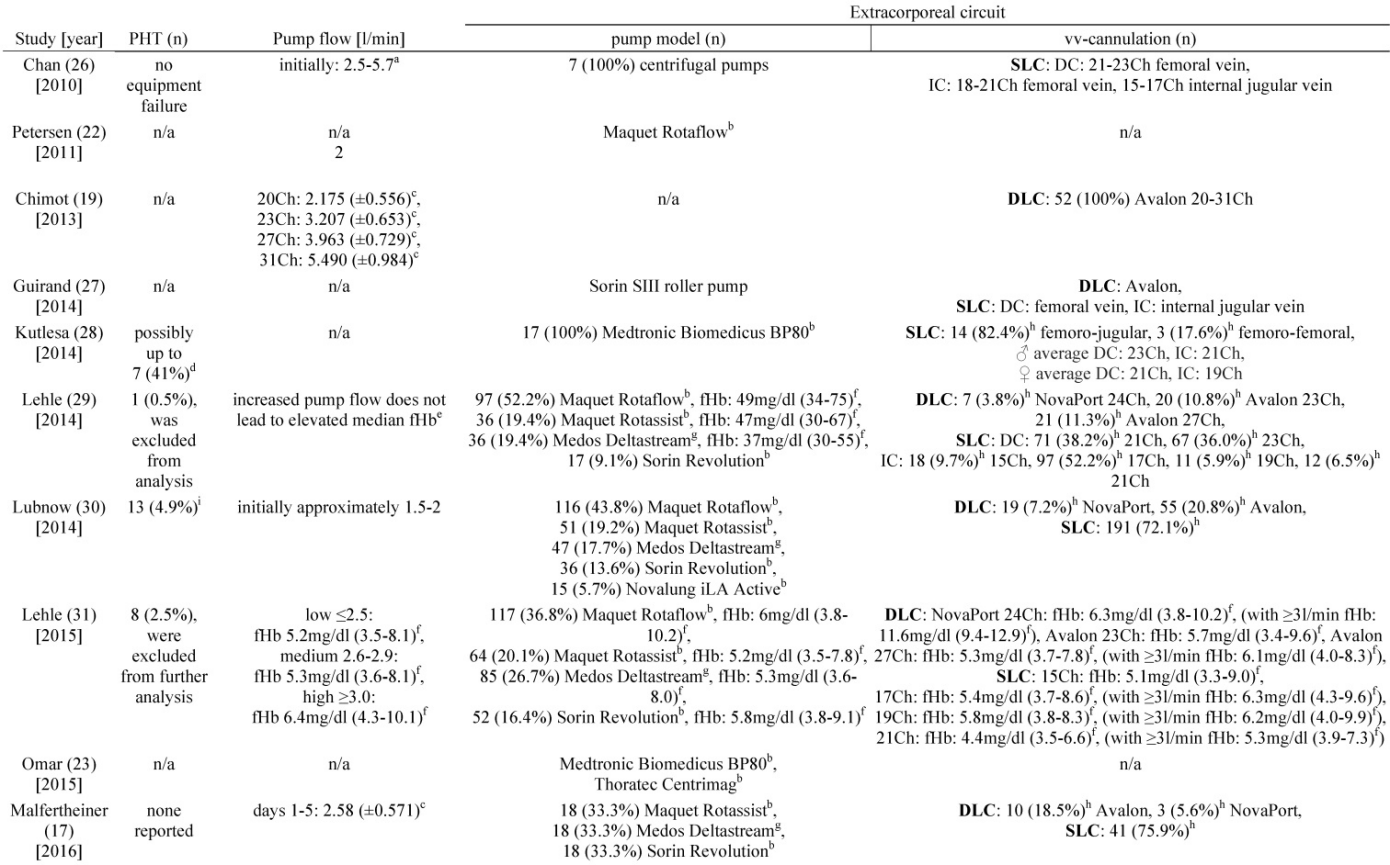

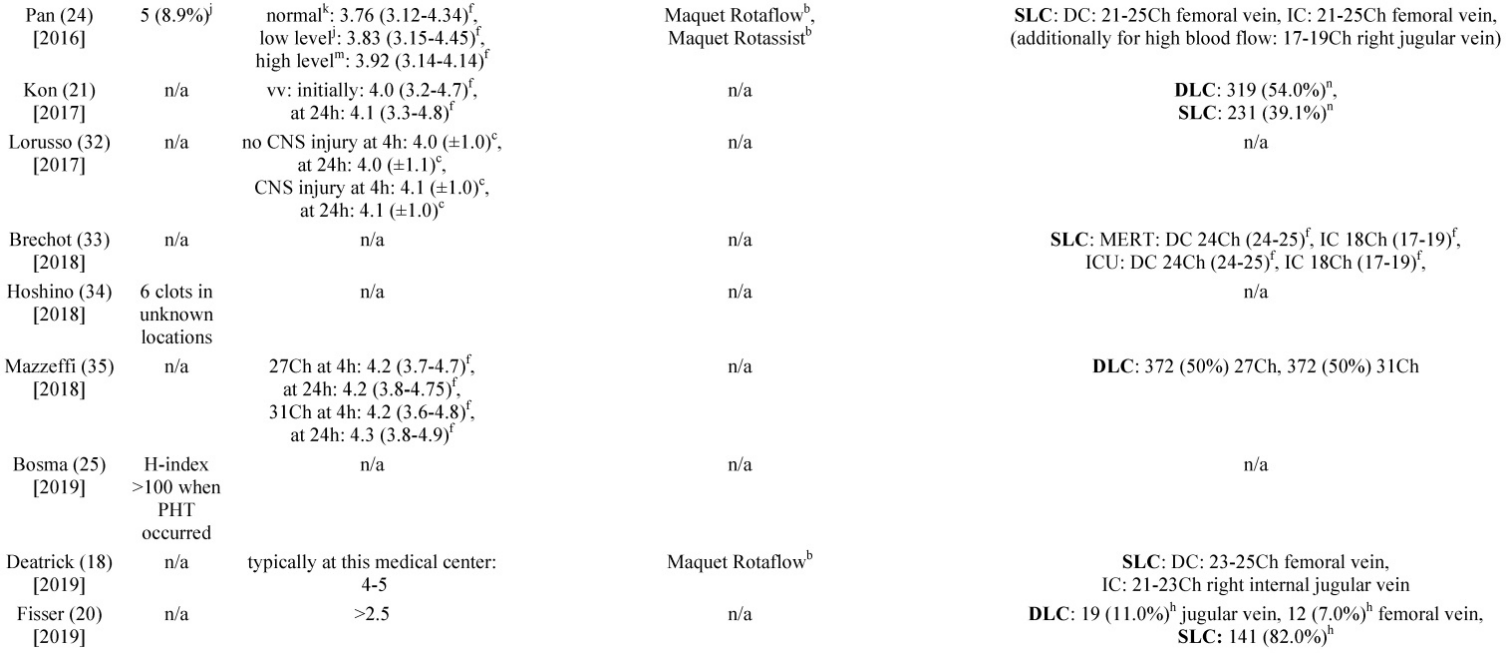
Pump head thrombosis, pump flow und extracorporeal circuit

a: range, b: centrifugal pump, c: standard deviation, d: pump head or entire system exchanged, e: independent of pump model and inflow cannula (DLC or SLC), f: interquartile range, g: diagonal pump, h: percentages were calculated with the given data, i: calculated for all 265 patients, j: calculated for 56 vvECMO runs, k: fHb-plasma level <10mg/dl, l: fHb-plasma level 10-50mg/dl, m: fHb-plasma level >50mg/dl, n: cannulation only documented for 550 of the 591 vvECMO runs. **Abbreviations:** PHT: pump head thrombosis, vv: veno-venous, SLC: single-lumen cannula, DC: drainage cannula, Ch: Charrière, IC: inflow cannula, n/a: not applicable, DLC: dual-lumen cannula, fHb: plasma free hemoglobin, MERT: mobile ECMO retrieval team, ECMO: extracorporeal membrane oxygenation, H-index: hemolysis index.
